# The equivalence of different types of electric pulses for electrochemotherapy with cisplatin − an *in vitro* study

**DOI:** 10.2478/raon-2024-0005

**Published:** 2024-02-21

**Authors:** Maria Scuderi, Janja Dermol-Cerne, Janez Scancar, Stefan Markovic, Lea Rems, Damijan Miklavcic

**Affiliations:** Faculty of Electrical Engineering, University of Ljubljana, Ljubljana, Slovenia; Department of Environmental Sciences, Jožef Stefan Institute, Ljubljana, Slovenia; Jožef Stefan International Postgraduate School, Ljubljana, Slovenia

**Keywords:** electrochemotherapy, electroporation, cisplatin uptake, phenomenological model, equivalent pulse parameters

## Abstract

**Background:**

Electrochemotherapy (ECT) is a treatment involving the administration of chemotherapeutics drugs followed by the application of 8 square monopolar pulses of 100 μs duration at a repetition frequency of 1 Hz or 5000 Hz. However, there is increasing interest in using alternative types of pulses for ECT. The use of high-frequency short bipolar pulses has been shown to mitigate pain and muscle contractions. Conversely, the use of millisecond pulses is interesting when combining ECT with gene electrotransfer for the uptake of DNA-encoding proteins that stimulate the immune response with the aim of converting ECT from a local to systemic treatment. Therefore, the aim of this study was to investigate how alternative types of pulses affect the efficiency of the ECT.

**Materials and methods:**

We performed *in vitro* experiments, exposing Chinese hamster ovary (CHO) cells to conventional ECT pulses, high-frequency bipolar pulses, and millisecond pulses in the presence of different concentrations of cisplatin. We determined cisplatin uptake by inductively coupled plasma mass spectrometry and cisplatin cytotoxicity by the clonogenic assay.

**Results:**

We observed that the three tested types of pulses potentiate the uptake and cytotoxicity of cisplatin in an equivalent manner, provided that the electric field is properly adjusted for each pulse type. Furthermore, we quantified that the number of cisplatin molecules, resulting in the eradication of most cells, was 2−7 × 10^7^ per cell.

**Conclusions:**

High-frequency bipolar pulses and millisecond pulses can potentially be used in ECT to reduce pain and muscle contraction and increase the effect of the immune response in combination with gene electrotransfer, respectively.

## Introduction

Electrochemotherapy (ECT) is a highly effective local treatment used in clinics to treat superficial tumors, specifically various types of skin tumors when standard treatments such as surgery, chemotherapy, and radiotherapy are not sufficient or applicable.^1,2^ Over the past decade, ECT has also been successfully used for the treatment of deep-seated tumors, including tumors in the liver, bone, and pancreas.^3,4,5,6,7,8,9^

ECT essentially consists of two main steps.^10,11^ First, a chemotherapeutic drug is injected intratumorally or intravenously. Second, short high-intensity electric pulses that result in cell membrane electroporation are delivered to the tumor. Electroporation transiently increases the cell membrane permeability through the formation of pores/defects in the membrane and enhances the intracellular uptake of the chemotherapeutic drug. The drugs most often used in ECT are bleomycin and cisplatin, which kill cancerous cells by acting on DNA but poorly permeate the cell membrane.^1,12^ Electroporation potentiates the uptake, and consequently the cytotoxicity, of bleomycin by several hundred to thousand folds and of cisplatin by several ten folds compared to nonelectroporated controls.^13,14,15^ In addition to increased intracellular drug delivery, drug entrapment due to the blood flow modifying effect of electric pulses^16^, the vascular disrupting effect^17,18^ and immune system response^19,20^ were identified to critically contribute to the success of ECT.^21^

Electroporation can be achieved with a wide range of pulse parameters (pulse shape, polarity, duration, amplitude, number, repetition rate, etc.). In ECT, conventionally 8 square monopolar pulses of 100 μs duration at a repetition frequency of 1 Hz or 5000 Hz are applied.^10,11,22^ However, the use of 100 μs long pulses causes pain and muscle contractions^23,24^ in the patient during the treatment. Furthermore, muscle contraction might lead to the displacement of the electrodes resulting in undertreatment^25^ and in potential harm for the vital structures when treating deep-seated tumors.^26^ Thus, there is a need to use local or general anesthesia and muscle relaxants and, when performing ECT of deep-seated tumors in proximity to the heart, the pulses need to be synchronized with the heart rhythm.^27,28,29,30,31^ To overcome these drawbacks, recent studies suggest the use of bursts of short high-frequency bipolar pulses (1–10 μs pulse duration), which minimize pain and muscle contractions.^23,32,33^ Such pulses are already used for the ablation of tumors^34^ and cardiac tissue^35,36,37^ by irreversible electroporation. Furthermore, *in vitro* and *in vivo* studies show that high-frequency bipolar pulses can potentially be used in ECT.^38,39^ Recent reports demonstrated the safety, tolerability, and efficacy of using high-frequency bipolar pulses for the treatment of cutaneous tumors with ECT.^40,41,42^

In ECT preclinical and clinical studies have shown that immune response critically contributes to tumor eradication.^19,43,44^ Thus, ECT has been tested in combination with gene electrotransfer (GET) which delivers protein-encoding DNA into tumor cell/tissue to induce immune stimulation.^45,46,47^ Even if the combined ECT+GET treatment was applied only to some of the cutaneous metastases, this combination successfully evoked a systemic immune response and in some cases succeeded in producing a partial response or complete response of distant, non-treated nodules (i.e., abscopal effect).^48^ GET, which is also based on electroporation, is traditionally achieved by the application of millisecond-duration electric pulses as it is believed that different transmembrane pathways/mechanisms are involved in chemotherapeutic vs. pDNA transport.^49^ When ECT is used in combination with GET traditionally two different types of pulses would be necessary (conventional 8 × 100 μs pulses for ECT and millisecond duration pulses for GET).

Changing the conventional 8 × 100 μs pulses to an alternative type of pulse such as high-frequency bipolar pulses or millisecond pulses could thus be advantageous in ECT. However, it is not well understood whether the use of alternative types of pulses would compromise the efficiency of the ECT treatment. Recently, *in vitro* study by Radzevičiūte *et al.*^50^ and *in vivo* study by Novickij *et al.*^51^ demonstrated that pulses of sub-microsecond duration can be as effective as the conventional pulses for ECT with bleomycin. Moreover, *in vitro* study by Vižintin *et al.*^52^ demonstrated that sub-microsecond pulses can be as effective as the conventional 8 × 100 μs pulses for ECT with cisplatin. The study^52^ also quantified the number of internalized cisplatin molecules needed for decreasing cell survival.

In this study, we expanded upon Vižintin *et al.*^52^ and investigated how high-frequency bipolar pulses and millisecond duration pulses affect the uptake and cytotoxicity of cisplatin compared with conventional 8 × 100 μs pulses. We performed *in vitro* ECT experiments, quantified the number of internalized cisplatin molecules and determined cisplatin cytotoxicity for the selected types of pulses. Our results demonstrated that the tested types of pulses resulted in equivalent drug uptake and cytotoxicity, provided that the electric field strength was adjusted for each pulse type separately. The quantified number of internalized cisplatin molecules producing a cytotoxic effect was in agreement with the Vižintin *et al.*^52^ study. We also tested a simple phenomenological model to describe the uptake of cisplatin molecules following cell exposure to different types of pulses. We discussed how the development of such models describing electroporative cisplatin uptake could provide a tool for treatment planning, using arbitrary types of pulses.

## Materials and methods

### Cell preparation

We used Chinese hamster ovary cell line (CHO-K1; cat. no. 85051005, European Collection of Authenticated Cell Cultures, United Kingdom). Cells were grown in 25 cm^2^ culture flasks (no. 90026, TPP, Switzerland) for 2–4 days in an incubator at 37°C, in a humidified atmosphere with 5% CO_2_. CHO cells were cultured in Ham-F12 growth medium (cat.no. N6658, Sigma Aldrich, Germany) supplemented with 10% fetal bovine serum (cat. No. F9665, Sigma Aldrich, Germany), L-glutamine (cat. No. G7513, Sigma Aldrich, Germany), antibiotics penicillin/streptomycin (cat.no. P0781, Sigma Aldrich, Germany), and gentamycin (cat.no. G1397, Sigma Aldrich, Germany). The cell suspension was prepared by detaching the cells in the exponential growth phase with 10× trypsin-EDTA (cat. no. T4174, Sigma Aldrich, Germany), diluted 1:9 in Hank’s basal salt solution (cat. no. H4641, Sigma Aldrich, Germany). After no more than 2 minutes, trypsin was inactivated by adding Ham-F12, and cells were transferred to a 50 ml centrifuge tube. Then, the cells were centrifuged (5 min, 180 g, 21°C) and re-suspended in Dulbecco’s Modified Eagle Medium (DMEM, cat. no. D5671, Sigma-Aldrich, Missouri, United States) supplemented with 10% FBS (cat. no. F9665, Sigma-Aldrich), 2.0 mM L-glutamine, 1 U/ml penicillin-streptomycin and 50 μg/ml gentamycin. The CHO cells were re-suspended at concentrations of 4 × 10^6^ cells/ml (permeability and survival experiments for determination of the optimal electric field) and 4.2 × 10^6^ cells/ml (for the clonogenic assay experiments and intracellular platinum concentration experiments).

### Pulse parameters and pulse application

**FIGURE 1.**
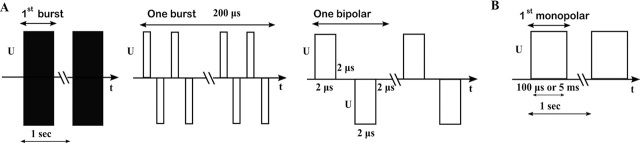
**(A)** 50 × 50 HF pulses. From left to right: 50 bursts were applied with a repetition frequency of 1 Hz; one burst with 200 μs total pulse on time and consisted of 50 bipolar pulses; one bipolar pulse of amplitude U consisted of a 2 μs long positive pulse, and a 2 μs long negative pulse (both of voltage U) with a 2 μs long interpulse delay. **(B)** 8 × 100 μs or 8 × 5 ms monopolar pulse of amplitude U and pulse duration of 100 μs or 5 ms were applied with a repetition frequency of 1 Hz

Three different types of pulses were used to perform experiments. For brevity, we refer to the three types of pulses used as 50 × 50 HF pulses, 8 × 100 μs pulses, and 8 × 5 ms pulses, and they are described in detail as follows. (i) The first type consisted of high-frequency bipolar pulses, specifically 50 bursts with a repetition frequency of 1 Hz. Each burst contained 50 short bipolar pulses having a pulse duration of 2 μs for the positive as well as for the negative pulse. The interpulse delay between consecutive bipolar pulses was 2 μs (Figure 1A). These high-frequency bipolar pulses were delivered by the pulse generator L-POR V0.1 (mPOR, Slovenia) at various voltages ranging from 80 V to 320 V with a step of 40 V. (ii) The second type of pulses consists of eight 100 μs monopolar pulses delivered at a repetition frequency of 1 Hz. These pulses were delivered by a prototype pulse generator based on H-bridge digital amplifier with 1 kV MOSFETs developed in our lab and described previously.^39^ The voltage of these pulses varied from 80 V to 320 V with a step of 40 V, Figure 1B. (iii) The third type consists of eight 5 ms long monopolar pulses, delivered at a repetition frequency of 1 Hz. These pulses were delivered by BTX Gemini X2 pulse generator (Harvard Apparatus, USA). Note that the pulse on time (the time when the voltage was different than zero) was 20 ms for 50 × 50 HF pulses, 800 μs for 8 × 100 μs, and 40 ms for 8 × 5 ms pulses. The voltage of these pulses varied from 80 V to 160 V with a step of 20 V (Figure 1B). The electric pulses were applied to cells in suspension placed in 2 mm aluminium cuvette. To ensure the quality of the delivered pulses the voltage and the current were monitored in all experiments with an oscilloscope Wavesurfer 422, 200 MHz, a differential voltage probe ADP305, and a current probe CP030 (from LeCroy, USA), according to the recommendations.^53^

### Permeability and survival curves for determination of the optimal electric field strength

To select the optimal electric field strength, i.e., where the highest cell membrane permeability and highest survival are achieved, we determined the so-called permeability and survival curves for each of the tested types of pulses. The selected optimal electric field strength was later used in the experiments with cisplatin.

To determine the permeability curve, the cell suspension was mixed with YO-PRO-1 iodide (cat. no Y3603, Thermo Fisher Scientific, Massachusetts, USA) to a final concentration of 1 μM. 150 μl of cells-YO-PRO-1 mixture was transferred in a 2 mm aluminum cuvette and then pulses were applied. 20 μl of the treated sample was transferred to a 1.5 ml centrifuge tube. Three minutes after pulse delivery the treated sample was diluted in 150 μl of DMEM and vortexed. The uptake of YO-PRO-1 was measured on the flow cytometer (Attune NxT; Life Technologies, Carlsbad, CA, USA). Cells were excited with a blue laser at 488 nm, and the emitted fluorescence was detected through a 530/30 nm band-pass filter. For each measurement, we acquired 10,000 events. Single cells were separated from all events by gating. Obtained data were analyzed using the Attune NxT software. The percentage of permeabilized cells was determined from the histogram of YO-PRO-1 fluorescence (see Supplementary Info S1).

To determine the survival curve, 150 μl of cell suspension was transferred to a 2 mm aluminum cuvette and then pulses were applied. 20 μl of the treated sample was transferred to a 1.5 ml centrifuge tube, and 25 minutes after pulse delivery, the samples were diluted in 380 μl Ham-F12. The cell suspension was gently mixed and 100 μl were transferred per well of a 96-well plate in triplicates. After 24 h of incubation in a humidified atmosphere at 37°C and 5% CO_2_, the MTS assay (CellTiter 96® AQueous One Solution Cell Proliferation Assay (MTS), Promega, USA^54^) was performed. The MTS assay was used to quantify the number of viable cells by evaluating their metabolic activity by measuring the formazan absorbance at 490 nm on a microplate reader (Tecan Infinite 200 pro; Tecan, Grödig, Austria). Cell survival was determined by first subtracting the background (signal from blank wells containing medium without cells) from all measurements and then normalizing the absorbance of the treated samples to the absorbance of the control sample.

### Clonogenic assay

We determined cisplatin cytotoxicity in combination with electroporation pulses using the clonogenic assay which is based on the ability of a single cell to divide and grow into colonies.^55^ On the day of the experiment, saline solution was used to dilute cisplatin (Cisplatin Kabi, 1 mg/ml, Fresenius Kabi, Germany or Cisplatin Accord, 1 mg/ml, Accord, UK) and prepare working solutions. The final concentrations of cisplatin during electroporation were 0 μM, 10 μM, 30 μM, and 50 μM. First, 150 μl of cell suspension with added specific cisplatin concentration was transferred to a 2 mm aluminium cuvette and then the suspension was exposed to the selected type of pulses of the optimal electric field as described in the subsection *Permeability and survival curves for determination of the optimal electric field strength*. Control samples received no pulses and no cisplatin. 25 minutes after pulse delivery, 5 μl of each sample (treated and control) was diluted in 495 μl Ham-F12 and mixed. Then, the number of cells in suspension was counted using Countess 3 Automated Cell Counter (Thermo Fisher Scientific). For each sample, specific dilutions were prepared to transfer ~100 live cells in each well of a 6-well plate in triplicates. Note that a higher number of cells was plated for the treated samples compared to the control sample^55^ to compensate for the cells which died immediately after the treatment. After 7 days of incubation at 37 °C and humidified atmosphere with 5% CO_2_, the growth medium was removed. The attached cells/colonies were fixed with methanol and stained with crystal violet for 10 minutes and washed. The colonies in each well were manually counted. First, we determined the average plating efficiency by dividing the number of counted colonies with the number of plated cells (specific for each experimental group). Then we determined cell survival by normalizing the average plating efficiency to the plating efficiency of the control sample.

### Determination of intracellular platinum concentration

Cells were prepared and treated with electric pulses in the presence of different concentrations of cisplatin, as described in previous section 25 minutes after pulse delivery, each sample was diluted in Ham-F12, centrifuged, and washed twice. The cell pellet was separated from the supernatant and the intracellular concentration of platinum was analyzed using inductively coupled plasma mass spectrometry (ICP-MS). To aid sample digestion, 0.1 ml of H_2_O_2_ and 0.1 ml of HNO_3_ (both from Merck, Darmstadt, Germany), were added to the cell pellets. The tubes were then sealed with caps and Teflon tape and left overnight at 80°C. Following digestion, 1.8 ml of Milli-Q water (Direct-Q 5 Ultrapure water system; Merck Millipore, Massachusetts, USA) was added. The platinum content in the samples was then measured using ICP-MS (7900 ICP-MS; Agilent Technologies, California, USA) with ^193^Ir (Merck, Darmstadt, Germany) used as an internal standard during the measurement.

To determine the amount of Pt per cell, the number of cells in the pellet was divided with the measured Pt in the cell pellet of each sample. To assess the number of cisplatin molecules per cell, it was assumed that 1 mol of Pt is equivalent to 1 mol of cisplatin. Control samples (not electroporated cells that were not incubated with cisplatin) were used for blank subtraction for all cisplatin-treated samples. To reduce cross-contamination of the instrument during the measurement, a mixture containing 1% HNO_3_ and 1% HCl (Merck, Darmstadt, Germany) was used as a rinse between the sample runs.

### Statistical analysis

Statistical analysis was performed using Prism 9.4.1 (GraphPad Software, USA). For permeability and survival experiments we performed oneway ANOVA if the normality test passed, or the ANOVA on ranks if the normality test failed with the Shapiro-Wilk test. For cisplatin cytotoxicity and cisplatin uptake experiments, we performed two-way ANOVA (independent variables: cisplatin concentration and pulse type). The normality test passed with the D’Agostino-Pearson test. Statistically significant difference was analyzed with respect to the control group (no cisplatin, no pulses) for all experiments. In the figures, the asterisk (*) indicates p < 0.05.

### Modeling

**FIGURE 2.**
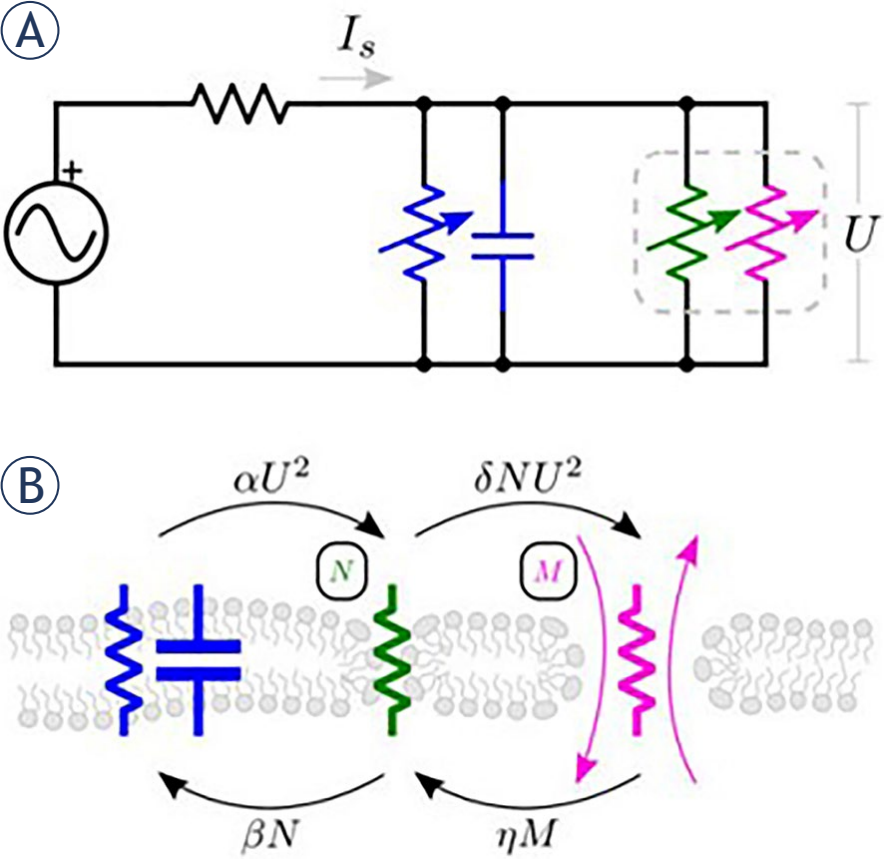
Schematic of the model that describes electroporation and molecular transport. **(A)** The equivalent circuit, which considers electroporation (membrane pore/defect formation) to be a two-step process, as depicted in **(B).** The blue capacitance and resistance represent the intact cell membrane. When the electric field is applied, the cell membrane becomes permeable first to small ions, indicating the first porous state (N) of the membrane represented by green resistance. Then the membrane becomes permeable to small molecules, indicating the second porous state (M) of the membrane represented by magenta resistance. Reproduced from Sweeney *et al.*56 with permission.

To model the intracellular uptake of cisplatin molecules, we used the phenomenological model developed by Sweeney *et al.*^56^, which is based on an equivalent circuit (Figure 2). The model considers a spherical cell exposed to electric pulses between parallel plate electrodes (homogenous electric field distribution), describes pores/defects formation as a two-state process, and considers diffusion as the only mechanism of transmembrane molecular transport. The model does not describe the cell spatially thus, the parameters used in the model are representative of the whole cell.

The source current *I_s_* describes how the electric field induces a voltage on the cell membrane. This induced transmembrane voltage *(U)* and *I_s_* are described by:

[1] $$\frac{\mathrm{dU}}{d\tau }=I_{s}-U\left(1+\gamma \left(N+M\right)\right)$$

[2] $$I_{s}=\frac{\tau_{\mathrm{RC}}\sigma_{\mathrm{EXT}}hE_{0}}{U_{0}\varepsilon_{m}}$$

The term *σ_EXT_* is the conductivity of the electroporation medium, h is the membrane thickness, *E_0_* is the applied electric field, *U_0_* is the electroporation threshold voltage and *ɛ_m_* is the membrane dielectric permittivity.

The increase of the transmembrane voltage results in the formation of small pores/defects (first porous state *N*) allowing the transmembrane transport of ions only. The presence of ionic currents decreases the value of the transmembrane voltage. The formed pores/defects can expand radially to allow transmembrane uptake of small molecules (second porous state *M*). The transport of small molecules into the cell is assumed to be governed by diffusion only, i.e., due to a concentration gradient between the extracellular and intracellular environment. The first porous state *N*, where only small ions can pass through the cell membrane, and the second porous state M, where also small molecules can pass through the cell membrane, are described by equations [3] and [4], respectively. The normalized intracellular concentration *(X)* of a selected molecule (here cisplatin) that crosses the membrane in the *M* state is described by equation [5]. All the differential equations are expressed as a function of the normalized time *(τ)*, which is defined as *τ = t/τ_RC_*, where t is the real-time and *τ_RC_* is the membrane charging time constant.

[3] $$\frac{dN}{d\tau }=\alpha U^{2}-\delta U^{2}N-\beta N+\eta M$$

[4] $$\frac{dM}{d\tau }=\delta U^{2}N-\eta M$$

[5] $$\frac{dX}{d\tau }=\xi M\left(1-X\right)$$

We implemented the model using a custom script in Matlab 2019b (MathWorks, Natick, MA, USA) and verified that the model in its original form reproduces the published results.^56^ The model was developed using the same cell line as in this study (CHO-K1) but based on quantitative measurements of propidium iodide uptake. Therefore, we modified the parameters related to the transported molecule, in our case cisplatin. Specifically, we changed the value of the solute radius *(ρ_s_)*, solute diffusivity *(D)*, the permeability coefficient *(ξ)*. The latter was determined by:

[6] $$\xi =\frac{3H\left(\lambda_{m}\right)D\;\tau_{RC}}{rh}$$

The term *H(λ_m_)* is the hindrance factor evaluated using the Renkin equation^57^, *D* is the diffusion constant of cisplatin, and r is the cell radius. We also adapted the conductivity of the electroporation medium *(σ)* to correspond to DMEM used in our experiments. The final model parameters are shown in Table 1. We performed calculations for pulse parameters used in our present and preceding^52^ studies: 50 × 50 HF pulses, 8 × 100 μs pulses, 8 × 5 ms pulses, and 1 × 200 ns pulse of 12.6 kV/cm and 25 × 400 ns pulses of 3.9 kV/cm applied at 10 Hz repetition frequency.

**TABLE 1.** Model parameters

**Parameter**	**Symbol**	**Value**	**Reference**
Electroporation threshold voltage	*U_0_*	258 mV	^56^
Membrane thickness	*h*	5 nm	^56^
Cell radius	*r*	7.5 μm	^56^
Membrane time constant	*τ_RC_*	1 µs	^56^
Membrane permittivity	*ɛ_m_*	12 × 8.85 × 10^−12^ F/m	^56^
Solute radius	*ρ_s_*	0.58 nm	^58^
Defect radius	*ρ_d_*	0.8 nm	^56^
Solute radius/Defect radius	*λ_m_* = *ρ_s_*/*ρ_d_*	0.7250	^56^
Solute diffusivity	*D*	1.670 × 10^−9^ m^2^/s	^58,59^
Parameter in N formation rate	*α*	2 × 10^−6^	^56^
N relaxation rate	*β*	4 × 10^−8^	^56^
Relative permeabilzed conductance	*γ*	1 × 10^6^	^56^
Parameter in M formation rate	*δ*	1 × 10^−3^	^56^
M relaxation rate	*η*	4 × 10^−9^	^56^
Permeability coefficient	*ξ*	8.45 × 10^−4^	^56^
Electroporation medium conductivity	*σ*	1.4 S/m	^*^

* Measured conductivity of DMEM using a conductometer (Mettler Toledo, S230)

The output of the model is the time course of the intracellular concentration of cisplatin *(X_i_ = X × X_e_*, where *X_e_* is the extracellular concentration of cisplatin) following the application of the electric pulses. We calculated the number of intracellular cisplatin molecules at time 25 minutes using:

[7] $$N=X_{i}\frac{4}{3}\pi r^{3}N_{A}$$

where 4/3 *πr*^3^ is the average volume of a cell and *N_A_* is the Avogadro number. The number of cisplatin molecules obtained with the model was then compared with the corresponding experimental measurements.

## Results

### The optimal electric field strength for each type of pulse

First, we performed experiments to determine the optimal electric field strength for each of the three tested types of pulses, to be later used in the experiments with cisplatin. As the optimal electric field strength, we consider the one in which the highest permeability and highest cell survival are achieved.

**FIGURE 3.**
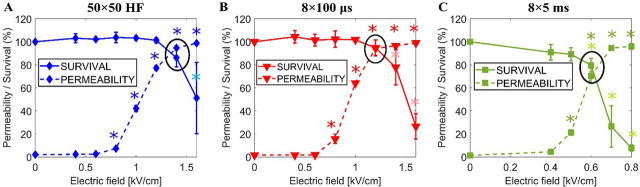
Cell survival (solid) and cell membrane permeability (dashed) as a function of the electric field when **(A)** 50 × 50 HF pulses; **(B)** 8 × 100 μs pulses; **(C)** 8 × 5 ms pulses are used. The chosen optimal electric fields are encircled. Each data point presents the mean ± standard deviation from 3–4 experiments. ^*^ = statistically significant differences from control (p < 0.05) performing one-way ANOVA if the normality test passed or otherwise ANOVA on ranks. The light blue, red, and green asterisks are related to survival experiments.

Figure 3 shows the experimentally determined permeability (dashed) and survival (solid) curves as a function of the applied electric field for A) 50 × 50 HF pulses, B) 8 × 100 μs pulses, and C) 8×5 ms pulses. Permeability curves show how the percentage of permeabilized cells increases with increasing electric field strength, whereas the survival curves show how the percentage of viable cells decreases with increasing electric field strength. The chosen optimal electric fields (i.e., highest permeability and highest survival) are 1.4 kV/cm for 50 × 50 HF pulses, 1.2 kV/cm for 8 × 100 μs pulses, and 0.6 kV/cm for 8 × 5 ms pulses.

### Cytotoxicity vs. the number of intracellular cisplatin molecules

**FIGURE 4.**
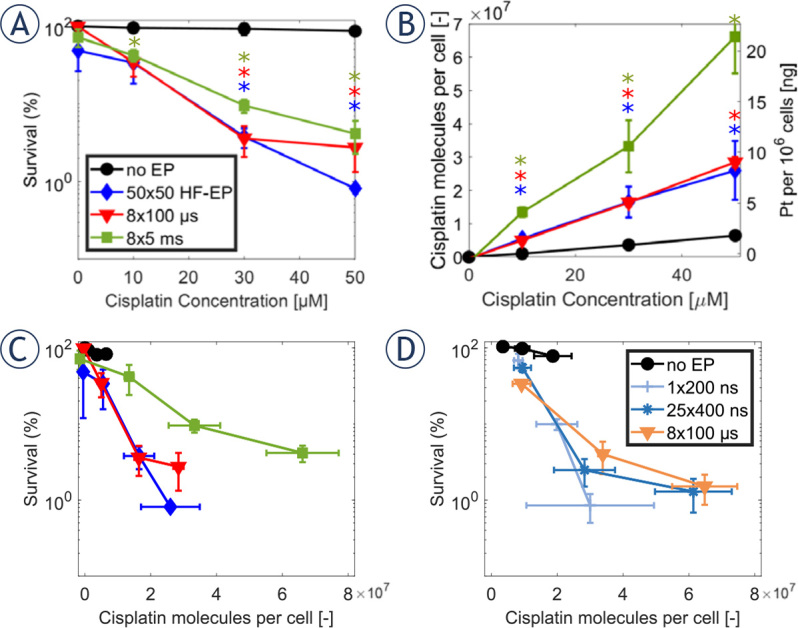
Cytotoxicity of cisplatin **(A)** and cisplatin molecules per cell **(B)** at different concentrations of cisplatin at a fixed electric field: 1.4 kV/cm for 50 × 50 HF pulses, 1.2 kV/cm for 8 × 100 μs pulses and 0.6 kV/cm for 8 × 5 ms pulses. Each data point presents the mean ± standard deviation from 3–4 experiments. ^*^= statistically significant differences from control (p < 0.05) performing twoway ANOVA test. The color of the asterisk corresponds to the line color for a specific type of tested pulse. Cell survival as a function of cisplatin molecules per cell in combination with electroporation **(C)** our experimental data and **(D)** experimental data replotted from Vižintin *et al.*^52^ with permission.

We next used the clonogenic assay to determine the cytotoxicity of cisplatin when exposing cells to the three types of pulses at their optimal electric field strength. Figure 4A shows how cell survival decreases as the extracellular concentration of cisplatin increases. In the absence of applied pulses, the tested cisplatin concentrations (0 μM, 10 μM, 30 μM, and 50 μM) do not affect cell viability (black curve). However, cytotoxicity is strongly potentiated with all three types of pulses, decreasing the cell survival to ~0.8% for 50 × 50 HF pulses, ~2.7% for 8 × 100 μs pulses, and ~4% for 8 × 5 ms pulses at the highest cisplatin concentration (50 μM) − note the logarithmic scale. Results for all three types of pulses are similar and are not statistically significantly different. Qualitatively similar results were obtained when measuring cell viability with the metabolic MTS assay (see Supplementary Info S2). However, as well known, the MTS assay reported better cell viability than the clonogenic assay for the same experimental conditions.^52,60^

We also measured the mass of intracellular Pt for each tested condition using ICP-MS and determined the average number of intracellular cisplatin molecules per cell, assuming that 1 mol of Pt is equivalent to 1 mol of cisplatin (Figure 4B). When no electric pulses are applied (black line), the number of cisplatin molecules increases slightly with increasing cisplatin concentration due to passive (i.e., diffusion), and active (i.e., membrane transporters^61,62,63^, endocytosis, pinocytosis, macrocytosis^64,65^) transport of cisplatin. However, when electric pulses are applied, the number of cisplatin molecules increases considerably. The greatest increase is observed for 8 × 5 ms pulses (up to 6.7 × 10^7^ at 50 μM). Roughly 2 times lower increase is observed for both 50 × 50 HF pulses and 8 × 100 μs pulses. There is a statistically significant difference between 8 × 5 ms pulses and the other two types of tested pulses when extracellular cisplatin concentration is 50 μM.

To determine the number of intracellular cisplatin molecules needed to achieve a cytotoxic effect, we combined the data from Figures 4A and 4B and plotted cell survival as a function of the number of cisplatin molecules in Figure 4C. Consistent with previous observations^13,52^, electroporation potentiates the cytotoxicity of cisplatin, as much lower survival is obtained with any of the three types of pulses compared with control for the same number of cisplatin molecules. The curves for 50 × 50 HF pulses and 8 × 100 μs pulses are almost overlapping, demonstrating that practically the same number of cisplatin molecules results in the same cytotoxic effect. Interestingly, in spite of two times higher Pt continent for 8 × 5 ms pulses the cytotoxicity is lower than when using 50 × 50 HF pulses or 8 × 100 μs pulses.

A previous study by Vižintin *et al.*^52^ used the same experimental protocols and analysis as here, but compared two other types of pulses with conventional 8 × 100 μs pulses, namely, 1 × 200 ns pulse of 12.6 kV/cm and 25 × 400 ns pulses applied at 10 Hz repetition frequency of 3.9 kV/cm. Their results are replotted in Figure 4D. This comparison demonstrates that the number of intracellular cisplatin molecules required to achieve a certain cytotoxic effect can be achieved with different types of pulses, if the electric field is properly adjusted.

### Modeling cisplatin uptake

**FIGURE 5.**
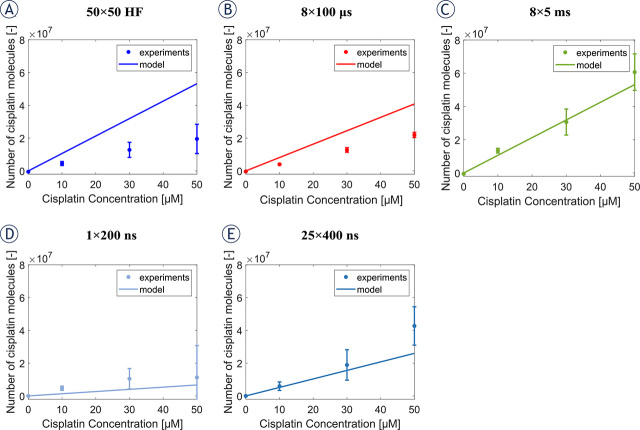
Comparison between the number of cisplatin molecules obtained experimentally (asterisks) and using the model (solid line) for **(A)** 50 × 50 HF pulses, **(B)** 8 × 100 μs pulses, **(C)** 8 × 5 ms pulses, **(D)** 1 × 200 ns pulses, and **(E)** 25 × 400 ns pulses. We used three different extracellular concentrations of cisplatin: 0 μM, 10 μM, 30 μM, and 50 μM.

Experimental data in previous section suggests that any type of pulses can be used for ECT, if it results in the same average number of internalized cisplatin molecules. Therefore, it would be useful to have a mathematical model for predicting the uptake of cisplatin molecules as a function of the pulse parameters. Figure 5 compares the measured uptake of cisplatin with prediction from a phenomenological model developed by Sweeney *et al.*^56^ for all types of pulses used in this and previous study.^52^ Note that the experimental data plotted in Figure 5 refer to the number of cisplatin molecules due to electroporation (i.e., we subtracted the uptake of cisplatin when no pulses were applied, black line Figure 4B). The model correctly predicts a proportional increase in the number of internalized molecules with increasing cisplatin concentration. The model also very well quantitatively predicts the number of cisplatin molecules experimentally obtained for 8 × 5 ms pulses, 1 × 200 ns pulses, and 25 × 400 ns pulses, but overestimates by ~2.5 and ~2 times the number of cisplatin molecules obtained for 50 × 50 HF pulses and 8 × 100 μs pulses, respectively. Nevertheless, for all pulse types, the model correctly captures the order of magnitude of the internalized cisplatin molecules.

## Discussion

In this study, we investigated how different types of pulses affect ECT *in vitro*. Specifically, we determined cisplatin uptake and cytotoxicity using CHO cells. We also tested a model that describes electroporation and the associated transmembrane molecular transport to predict the number of cisplatin molecules in an individual cell.

### Electroporation potentiates cisplatin uptake and cytotoxicity in a similar way for all tested types of pulses

Different types of pulses can be considered equivalent for electroporation when the electric field strength is adjusted for each type of pulses separately.^66^ We thus first determined, for each selected type of pulses, how the electric field strength affects the percentage of cells that become permeable to YO-PRO-1 and the percentage of cells that survive the exposure to electric pulses in the absence of cisplatin. YO-PRO-1 is a nucleic acid stain that allows rapid screening of permeabilized cells using flow cytometry. The size of YO-PRO-1 (630 Da) is somewhat larger, but nevertheless comparable to cisplatin (300 Da). Therefore, cells that become permeable to YO-PRO-1 are expected to also become permeable to cisplatin. Cell survival was measured 24 hours after electroporation with the metabolic MTS assay. An *in vitro* study by Peng *et al.*^67^ showed that 24 h is the adequate incubation time to measure cell survival following electroporation. The electric field strength, with the highest percentage of permeable cells and the highest percentage of viable cells, was 1.4 kV/cm for 50 × 50 HF pulses, 1.2 kV/cm for 8 × 100 μs pulses, and 0.6 kV/cm for 8 × 5 ms pulses. Consistent with Pucihar *et al.*^66^, longer pulses (8 × 5 ms) require lower electric fields to obtain a similar fraction of permeabilized cells than shorter pulses (8 × 100 μs). *In vitro* studies 38,39 have observed that a higher electric field is needed for high-frequency bipolar pulses than for monopolar pulses of 100 μs duration, with equivalent treatment time, to achieve a similar fraction of permeabilized cells. However, in this study, a similar electric field of 1.2 kV/cm and 1.4 kV/cm is required for 8 × 100 μs and 50 × 50 HF pulses, respectively, to achieve a similar fraction of permeabilized cells. This is due to the use of a higher number of bursts and bipolar pulses that reduce the required pulse amplitude. The average cell survival at the optimal electric field was for all pulse types above 80% and was not statistically significantly different from control except for 8 × 5 ms pulses. Overall, in terms of electroporation, all the three tested types of pulses of the optimal electric field strength can be considered equivalent.

We then performed *in vitro* ECT experiments. We measured the cytotoxicity and the uptake of cisplatin when exposing cells to all three types of pulses with the optimal electric field strength in the presence of different extracellular concentrations of cisplatin (0 μM, 10 μM, 30 μM, 50 μM). For all three types of pulses, we observed that an increase in cisplatin concentration increased cell cytotoxicity and intracellular uptake of cisplatin, which is in agreement with previous studies using mouse skin melanoma cells.^13,38,52,68^ Furthermore, the results for both cisplatin uptake and cytotoxicity were very similar for all three types of pulses, demonstrating that these pulse types can be considered, not only equivalent in terms of electroporation and transmembrane molecular transport, but also in terms of potentiation of cisplatin cytotoxicity and ECT. We also observed in Figures 4A and 4B that ~2 times higher amount of cisplatin molecules is needed for 8 × 5 ms pulses to achieve a similar cytotoxic effect as when using 50 × 50 HF pulses and for 8 × 100 μs pulses. Vižintin *et al.*^52^ reported that the structure of cisplatin is not affected when nanosecond and 8×100 μs pulses are used. However we cannot completely exclude that the structure of cisplatin might be affected by the higher amount of electrochemical reaction caused by ms pulses (we saw bubble formation during experiments) which might lead to a lower cytotoxic effect of cisplatin.^62,69,70^ Furthermore an *in vitro* study by Rols *et al.*^71^ showed that electroporation, using millisecond pulses, can induce long term micropinocytosis, thus cisplatin molecules might be entrapped in vesicles and not express their cytotoxic effect.

By combining the results on cisplatin cytotoxicity and cisplatin uptake, we were able to determine the number of internalized cisplatin molecules needed to achieve a cytotoxic effect. For all tested types of pulses, this number was in the range of 2−7 ×10^7^ cisplatin molecules per cell. Same range was obtained by Vižintin *et al.*^52^, who studied cisplatin cytotoxicity following exposure of cells to 1 × 200 ns and 25 × 400 ns pulses. Altogether the results suggest that, as long as the electric field is appropriately adjusted, different types of pulses can be used for potentiating cisplatin cytotoxicity, and consequently different types of pulses can be used for ECT. Thus, the 50 × 50 HF pulses, 8 × 100 μs, 8 × 5 ms pulses, 1 × 200 ns pulses, and 25 × 400 ns pulses of properly adjusted electric field strength can be considered for ECT.

### Clinical relevance

ECT has been demonstrated as a locally effective treatment of tumors of various histotypes.^72^ The consistent clinical success of ECT has been achieved through the meticulous development of pulse protocols, electrodes, and the publication of Standard Operating Procedures for cutaneous and subcutaneous tumors.^11^ It has been later demonstrated that also deep-seated tumors can be successfully treated by ECT provided the tumor is covered by sufficiently high electric fields either as intraoperative^5^ or percutaneous procedure.^31,73,74,75^ Accordingly, Standard Operating Procedures have been updated.^10^ With good success and acceptance by patients, larger tumors and patients with more extensive diseases were treated. Pain and muscle contraction-related high voltage pulse delivery became the most often reported side effects and alternative pulse waveforms that would maintain ECT efficacy but reduce pain and muscle contraction.^24^ In this respect, high-frequency bipolar short pulses ^33,34^ and also nanosecond pulses ^76,77^ were suggested. Furthermore, recent studies investigate how high-frequency bipolar pulses and nanosecond pulses affect ECT. Our previous *in vitro* study^38^ showed that similar cisplatin cytotoxicity is obtained by comparing high-frequency bipolar pulses and conventionally ECT pulses as soon as the electric field is properly adjusted. Lyons *et al.*^40^ have recently demonstrated the safety and efficiency of using high-frequency bipolar pulses in ECT using bleomycin for the treatments of 97 lesions of different histological subtypes of cutaneous malignancies in 25 patients. The authors observed an overall response rate of 86% (complete response rate 63.6%) three months after the treatment which is in agreement with the overall response rate of 85% (complete response rate 73.7%) determined in the ESOPE study of 2006^22^ and in a follow-on study using InspECT database in which the overall response is 85% (complete response rate 70%, partial response rate 15%).^72^ Thus, the data published by Lyons *et al.*^40^ showed that the use of high frequency bipolar pulses is equivalent regarding the overall response rate to the use of classical ECT pulses. Furthermore, the patients that were treated with local anesthesia showed excellent tolerability to the treatment. Thus, the use of high frequency bipolar pulses might possibly reduce the need to use general anesthesia during ECT shortening the overall hospital stay, reducing the time needed to recover and the costs and increasing the safety of the treatment.^78^

A study by Vižintin *et al.*^52^ compared the effects of conventional 8 × 100 μs pulses with nanosecond pulses on cisplatin uptake and cytotoxicity in cell lines *in vitro*. The authors showed that nanosecond pulses can be equally effective for ECT as conventional 8 × 100 μs pulses.^52,79^ These results are in agreement with *in vitro* studies on a tumor model, murine Lewis lung carcinoma (LLC1) cell line, by Radzevičiūte *et al.*^50^ and *in vivo* study by Novickij *et al.*^51^ which show that nanosecond pulses can be as effective as when using the conventional ECT pulses in ECT when using bleomycin as chemotherapeutic drug.

Considerable efforts are also focused on making ECT a systemic treatment by combining it with immunotherapy.^20,48,80,81^ Electrochemotherapy can induce immunogenic cell death through the release of damage-associated molecular patterns (DAMP) which serve as a signal to stimulate the immune system.^82,83,84^ Massive liberation of tumor antigens together with DAMPs can activate the antigen-presenting dendritic cells.^85,86^ Multiple studies in canine^87,88,89^, in mice^47^ and human patients^90^ have thus been testing ECT in combination with gene electrotransfer (GET) of plasmid DNA encoding for interleukin-12 (IL-12), which stimulates the immune system.^45,46,47^ Traditionally in GET millisecond duration pulses are used to deliver DNA into the cells.^91,92,93^ Thus, when ECT is used in combination with GET two different types of pulses 8 × 100 μs pulses and millisecond pulses are used, respectively. However, it might be beneficial to use the same type of pulses when ECT is combined with GET as this would allow the use of simpler pulse generators.

In order to capitalize on a significant body of clinical evidence we tested equivalence of such pulses by *in vitro* test, specifically determining the amount of chemotherapeutic drug delivery by electroporation pulses. In this way equivalent pulses are determined by delivering the same drug amount into cells, thus producing the same cytotoxicity. Therefore, the replacement of classical ECT pulses with nanosecond and high frequency bipolar pulses would be beneficial to reduce muscle contractions and pain, potentially avoiding the need for anesthetics and muscle relaxants during the treatment. Furthermore, using the same pulses (either long or short, or bipolar) for delivering cytotoxic drugs into the cells, as well as pDNA to achieve simultaneous gene electrotransfer, is an attractive idea that may be within reach.^94^

### Further development of mathematical models that can predict cisplatin uptake can help with electrochemotherapy treatment planning

For the success of ECT, all the tumor needs to be covered by an electric field of sufficient amplitude to permeabilize the cells/tissue, and a sufficient amount of chemotherapeutic drug is needed in the tumor.

In the Standard Operating Procedures all information related to the types of electrodes i.e., of fixed geometry, pulse parameters, and pulse generators^10,11,22^ which guarantee a complete coverage of the tumor are provided. However, to treat deep-seated tumors long needles with variable configuration electrodes are used.^95^ Thus, there is a need to determine the optimal position of the electrodes and the optimal pulse parameters for complete coverage of the tumor tissue. It is not trivial to determine how the electric field is distributed in biological tissues due to tissue-specific properties, the use of different types of electrode geometries, and pulse parameters. Treatment planning using numerical models helps clinicians to determine the optimal parameters to treat a specific tumor. Currently, in treatment planning a fixed threshold electric field is used to deem tissue permeabilized or not.^3,96^ However, just a high-enough electric field does not guarantee cell death as simultaneously a high-enough extracellular cisplatin concentration is needed to obtain enough internalized cisplatin molecules for cell death. Our model presents a missing link in the complete model for treatment planning, connecting the external electric field, the number of internalized cisplatin molecules, and cell death. The first building block for the multiscale model of tissue electroporation was published in Dermol-Černe *et al.* 2018^68^ where a model connecting extracellular and intracellular cisplatin concentration as a function of electric pulses was developed. Now, we went one step further and connected the intracellular cisplatin concentration with cell death.

A mathematical model that can also predict the uptake of molecules such as chemotherapeutics drugs (e.g., cisplatin and bleomycin) is needed to be determined for treatment planning for different pulse types is useful. Now we only need the final piece, and this is a model of cisplatin transport across the cell membrane as a function of electric pulses. Our previous study demonstrated that existing mechanistic models of electroporation have limited reliability for predicting the transmembrane transport of small molecules across a wide range of pulse parameters.^97^ We observed that the contribution of electrophoretic transport during pulse delivery is often overestimated. Therefore, we decided to test a phenomenological model developed by Sweeney *et al.*^56^ that neglects electrophoresis and takes into account only diffusion during and after pulse delivery. Furthermore, we selected this model since it is the simplest model that allows computation of the transmembrane transport of small molecules for arbitrary types of pulses. Indeed, the model is based on quantitative measurements of transmembrane transport of propidium iodide uptake (not cisplatin) induced by a single pulse of different pulse lengths (1, 10, 100, 1000 μs) and electric field strengths (1.7, 2.5, 3.2, 4 kV/cm) that are different from the ones used in our study. Despite its simplicity, and without any considerable model modifications (see the Modeling section), the model was able to predict the order of magnitude of cisplatin uptake for all tested pulse parameters. However, a basic parametric analysis (see Supplementary Info S3) showed that the results for different types of pulses depend in a different way on the model parameters. Therefore, a comprehensive parametric analysis and additional model development would be required, which is out of the scope of the present study (see the Clinical Relevance section). Based on the results obtained, we nevertheless expect that relatively simple models could be developed in the future as a tool for predicting cisplatin uptake.

### Limitation of the study

The drawback of our study is the use of only one cell line i.e., the Chinese hamster ovary cells (CHO-K1) non-cancerous cells to perform *in vitro* ECT experiments. It was observed *in vitro* that cancer cells behave differently than normal cells.^98^ However, an *in vitro* study published by Vižintin *et al.*^52^ showed a similar platinum uptake when using CHO-K1 cells or mouse skin melanoma B16-F1 cells and applying 8 × 100 μs pulses. Thus, we expect that the observed equivalence of different pulse types observed in CHO-K1 cells would also be observed in different cancer cells.

It has been shown that the immune system plays an important role in the efficiency of ECT. Electroporation can potentiate the cytotoxicity and uptake of cisplatin but can also stimulate the immune response by releasing damage-associated molecular patterns (DAMP). In our *in vitro* study we demonstrated the equivalence of different types of pulses on cisplatin uptake and cytotoxicity. Similarly, a recent *in vitro* study by Polajžer *et al.*^99^ showed the release of DAMP molecules (e.g. ATP, HMGBI, Calreticulin) albeit with some differences observed with different types of pulses.

We have focused on equivalent drug delivery to cells *in vitro* but this may be different *in vivo*. Thus, further studies in animals are needed to investigate the equivalence of different pulse types for drug entrapment by tumor blood flow modification, for the vascular disrupting action, and for the immune response in ECT.

## Conclusions

Our study focused on the effect of different types of electric pulses in ECT, particularly in terms of cisplatin uptake and cisplatin cytotoxicity, using CHO cells *in vitro*. We demonstrate that different types of pulses such as classical ECT pulses, high frequency bipolar pulses and millisecond pulses potentiate cisplatin uptake and cisplatin cytotoxicity. Moreover, we observed similar cisplatin uptake and cisplatin cytotoxicity when using different types of pulses i.e., considered equivalent provided that the electric field is properly adjusted. Thus, equivalent electric pulses such as high frequency bipolar pulses and nanosecond can potentially be used in ECT to reduce pain and muscle contraction while maintaining the same efficacy in cisplatin uptake and cisplatin cytotoxicity as when using the classical ECT pulses. Moreover, our results show that using one type of pulse when combining ECT with EGT is a concept that might be readily achievable considering the equivalent pulse parameters.

In addition, we experimentally determine the number of cisplatin molecules needed to achieve a cytotoxic effect which is in the range of 2−7 × 10^7^ cisplatin molecules per cell in agreement with previous study.^52^ We also used a mathematical model describing electroporation and transmembrane molecular transport, as a tool to predict the number of cisplatin molecules into individual cells when different types of pulses need to be tested. The future goal is to improve treatment planning by including a model that predicts the uptake of molecules such as cisplatin or bleomycin.

## Supplementary Material

Supplementary Material Details
