# Supplementary material for: The equivalence of different types of electric pulses for electrochemotherapy with cisplatin − an in vitro study
Source: Radiol Oncol. 2024 Feb 21;58(1):51–66. doi: 10.2478/raon-2024-0005 (PMC10878774; doi:10.2478/raon-2024-0005)
Supplement: Supplementary file 1 — Supplementary Material Details [file raon-2024-0005-sm.pdf]

The equivalence of different types of electric pulses for electrochemotherapy with cisplatin – an in vitro study

Maria Scuderi, Janja Dermol-Cerne, Janez Scancar, Stefan Markovic, Lea Rems, Damijan Miklavcic

Radiol Oncol 2024; 58(1): 51-66.

doi: 10.2478/raon-2024-0005

S1. Flow cytometer

We performed *in vitro* experiments using CHO-K1 cells to determine cell membrane permeability to YO-PRO-1 when different types of pulses are used. In figure S1, are shown the images acquired with the flow cytometer when 8×100 µs pulses at 1.2 kV/cm were used. First, single cells were acquired and separated from all events by gating, Figure S1 on the left. Then, the percentage of permeabilized single cells was determined from the histogram of YO-PRO-1 fluorescence by gating, Figure S1 on the right. Please note that the same gating was used also for 8×5 ms pulses and 50×50 HF pulses at different electric fields.

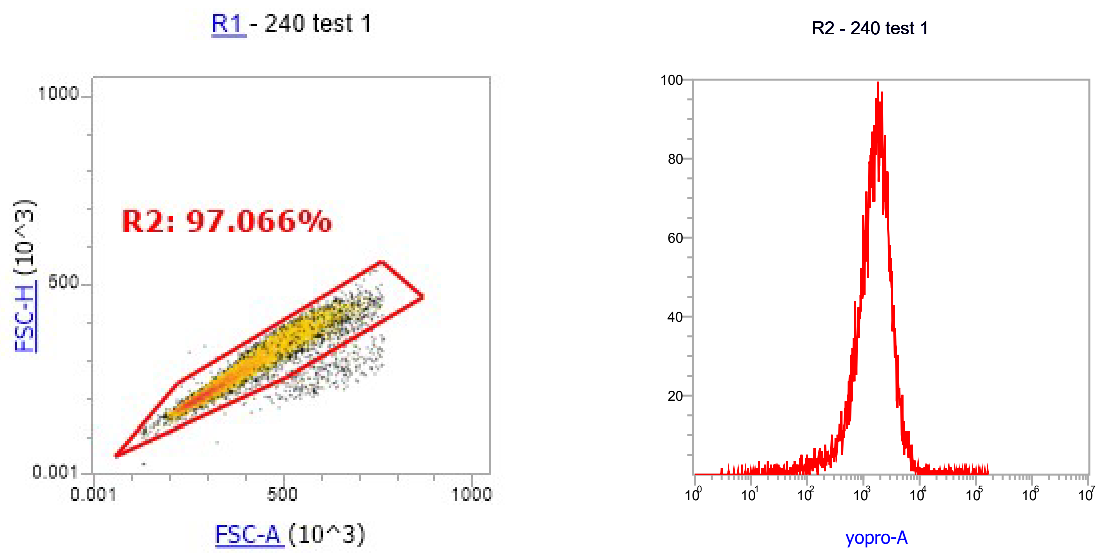

Figure S1. Cell membrane permeability to YO-PRO-1 measured by flow cytometry. On the left, the acquired single cells separated from all the events by gating. On the right histogram of YO-PRO-1 fluorescence.

S2. Cytotoxicity of cisplatin

We performed *in vitro* experiments using CHO-K1 cells to determine the cytotoxicity of cisplatin when exposing cells to different concentrations of cisplatin (0 μM, 10 μM, 30 μM, and 50 μM) and using a fixed electric field: 1.4 kV/cm for 50×50 HF pulses, 1.2 kV/cm for 8×100 μs pulses, and 0.6 kV/cm for 8×5 ms pulses. We measured cell survival with metabolic MTS assay. Figure S2 A, show how cell survival decreases increasing as the extracellular concentration of cisplatin increases. Combining cell survival (Figure S2 A) with the intracellular number of cisplatin molecules (Figure 4B) we determine the intracellular number of cisplatin molecules needed to achieve a cytotoxic effect, Figure S2 B. A similar cytotoxic effect (~40 % cell survival) is achieved with the three types of pulses.

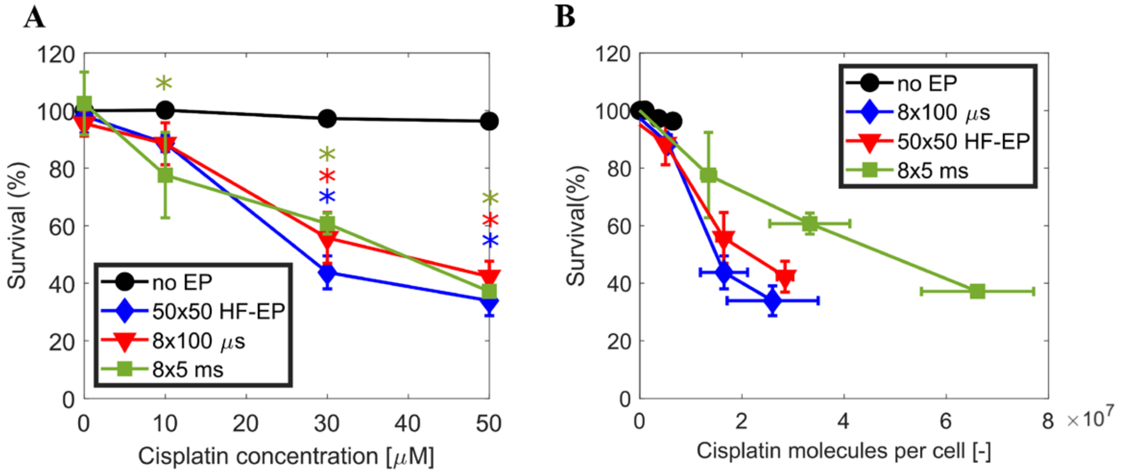

Figure S2. (A) Cytotoxicity of cisplatin when exposing cells to different concentrations of cisplatin at a fixed electric field: 1.4 kV/cm for 50×50 HF pulses, 1.2 kV/cm for 8×100 μs pulses, and 0.6 kV/cm for 8×5 ms pulses. (B) Cell survival as a function of cisplatin molecules per cell in combination with electroporation. *- statistically significant differences from control ($p<0.05$) performing 2-way ANOVA test. The color of the asterisk corresponds to the line color for a specific pulse type.

S3. Model: Basic sensitivity analysis

The phenomenological model developed by Sweeney et al. [56] qualitatively predicts the uptake of cisplatin molecules over a wide range of pulse parameters. The model showed that the number of cisplatin molecules increases increasing the extracellular concentration of cisplatin in agreement with the *in vitro* experiments of our study. The model can also very well quantitatively predict the number of cisplatin molecules in a cell due to electroporation for 1×200 ns pulses, for 25×400 ns pulses, and for 8×5 ms pulses. However, the model overestimate the number of cisplatin molecules for the other tested types of pulses. Thus, we decided to perform a basic parametric analysis to understand how the different types of pulses affect the model parameters. We multiplied or divided by a factor of 10 the parameters α, δ, β, η of the model. The parameters α, δ are related to membrane permeability, and β, η are related to membrane resealing. The basic parametric analysis shows that the number of cisplatin molecules for different types of pulses depends differently on the model parameters, Figure S3.

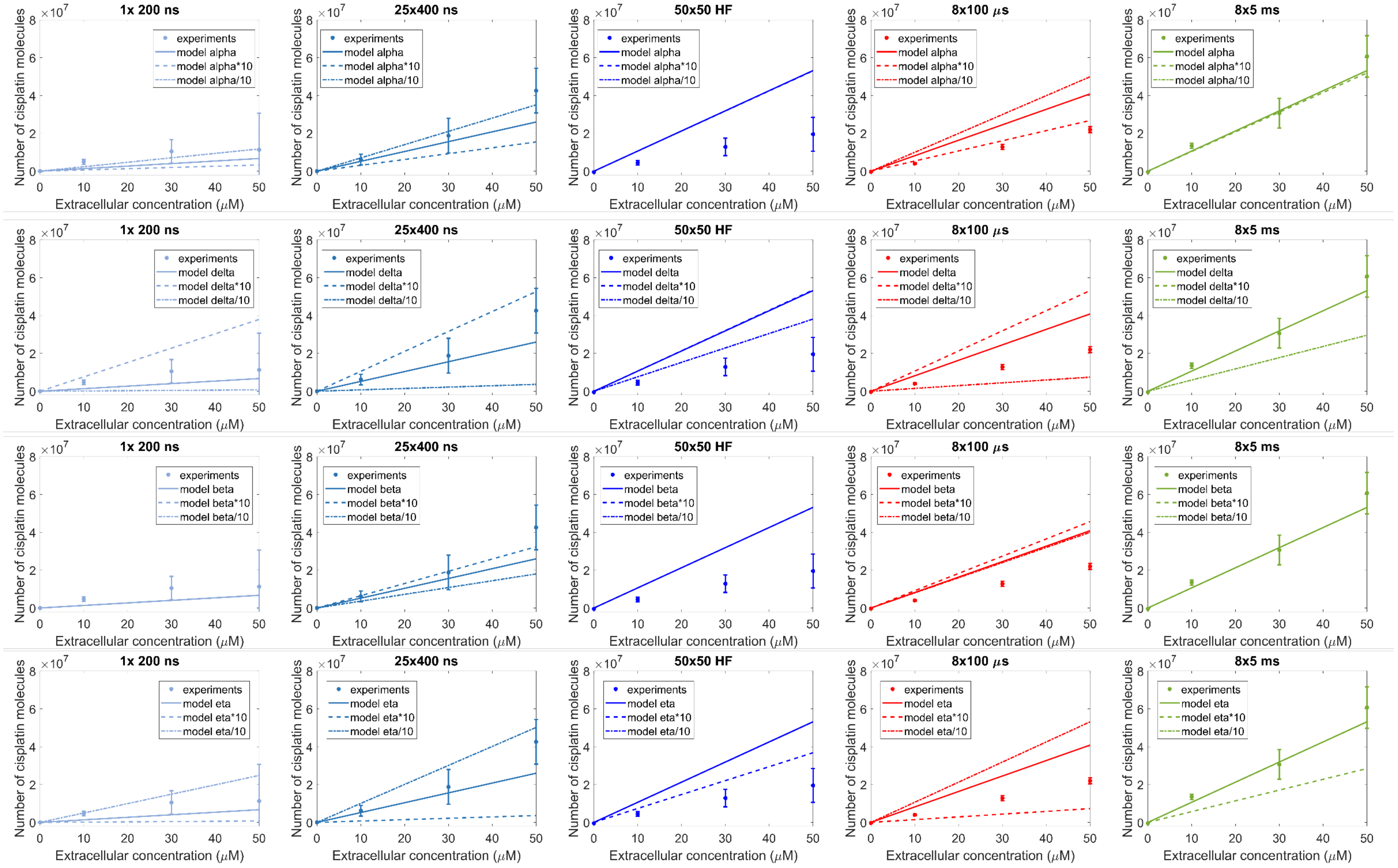

Figure S3. Basic parametric analysis investigating: α (first row), δ (second row), β (third row), η (fourth row) model parameters when using different types of pulses: 1×200 ns pulses, 25×400 ns pulses, 50×50 HF pulses, 8×100 μs pulses, and 8×5 ms pulses.
